# Ethyl 3-[2-(*p*-toluene­sulfonamido)phen­yl]acrylate

**DOI:** 10.1107/S1600536809034163

**Published:** 2009-09-05

**Authors:** Mei-Fang Jin, Bao-Yong Zhu

**Affiliations:** a Department of Chemical Engineering, Anyang Institute of Technology, 455000 Anyang, People’s Republic of China; b Department of Chemistry, Dezhou University, 253023 Dezhou, People’s Republic of China

## Abstract

In the title compound, C_18_H_19_NO_4_S, the two benzene rings form a dihedral angle of 52.2 (7)°. The crystal struture is stabilized by N—H⋯O hydrogen bonds, which link the molecules into dimers.

## Related literature

For functionalized carbon frameworks, see: Mukherjee *et al.* (2007 ▶). For sulfonamido compounds and their use in pharmaceuticals, see: Patchett *et al.* (1995 ▶). For a related structure, see: Senthil Kumar*et al.* (2006 ▶).
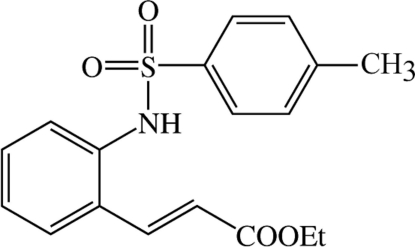


## Experimental

### 

#### Crystal data

C_18_H_19_NO_4_S
*M* *_r_* = 345.40
Triclinic, 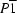

*a* = 8.001 (4) Å
*b* = 10.245 (5) Å
*c* = 11.402 (5) Å
α = 81.182 (5)°
β = 70.895 (4)°
γ = 86.604 (5)°
*V* = 872.7 (8) Å^3^
*Z* = 2
Mo *K*α radiation
μ = 0.21 mm^−1^
*T* = 291 K
0.46 × 0.43 × 0.38 mm

#### Data collection

Oxford Diffraction Gemini S Ultra diffractometer
Absorption correction: multi-scan (*SADABS*; Bruker, 2001 ▶) *T*_min_ = 0.91, *T*_max_ = 0.93
15343 measured reflections
3553 independent reflections
2784 reflections with *I* > 2σ(*I*)
*R*_int_ = 0.020

#### Refinement

*R*[*F* ^2^ > 2σ(*F* ^2^)] = 0.044
*wR*(*F* ^2^) = 0.147
*S* = 1.18
3553 reflections
219 parameters
H-atom parameters constrained
Δρ_max_ = 0.24 e Å^−3^
Δρ_min_ = −0.28 e Å^−3^

### 

Data collection: *CrysAlis Pro* (Oxford Diffraction, 2006 ▶); cell refinement: *CrysAlis Pro*; data reduction: *CrysAlis Pro*; program(s) used to solve structure: *SHELXS97* (Sheldrick, 2008 ▶); program(s) used to refine structure: *SHELXL97* (Sheldrick, 2008 ▶); molecular graphics: *ORTEP-3 for Windows* (Farrugia, 1997 ▶) and *CAMERON* (Pearce & Watkin, 1993 ▶); software used to prepare material for publication: *WinGX* (Farrugia, 1999 ▶).

## Supplementary Material

Crystal structure: contains datablocks global, I. DOI: 10.1107/S1600536809034163/bg2295sup1.cif

Structure factors: contains datablocks I. DOI: 10.1107/S1600536809034163/bg2295Isup2.hkl

Additional supplementary materials:  crystallographic information; 3D view; checkCIF report

## Figures and Tables

**Table 1:** Hydrogen-bond geometry (Å, °)

*D*—H⋯*A*	*D*—H	H⋯*A*	*D*⋯*A*	*D*—H⋯*A*
N1—H1⋯O3^i^	0.92	2.01	2.920 (2)	172

Symmetry code: (i) 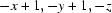.

**Fig. 1.**
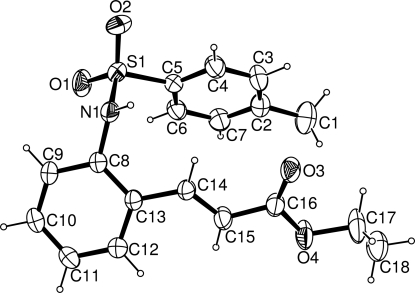
The molecular structure of (I), with the atom-numbering scheme. Displacement ellipsoide are drawn at 30% probability level.

**Fig. 2.**
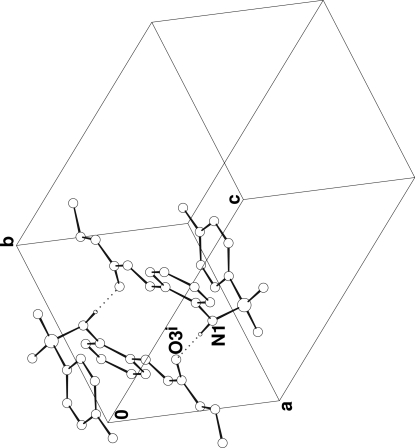
The dimeric structure of the title compound. Dotted lines indicate hydrogen bonds [Symmetry code: (i) = 1 - x, 1 - y, -z.]

**Fig. 3.**
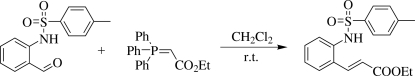
The formation of the title compound.

## supplementary crystallographic information

### Comment

Electron-deficient olefin, particularly α,β-unsaturated carbonyl compound, was used as a fundamental material to construct functionalized carbon frameworks (Mukherjee *et al.*, 2007). Sulfonamido is an important group in natural compounds and many pharmaceuticals (Patchett *et al.*, 1995). We selected *N*-(2-formylphenyl)(4-methylbenzene)sulfonamide and (ethoxycarbonylmethylene)triphenylphosphorane to synthesize a new compound formulated as C_18_H_19_N_1_O_4_S_1_ (I) with dimeric structures *via* hydrogen bonds.

The molecular structure of (I) is illustrated in Fig. 1. The geometry of the molecule is close to the related compound Ethyl 2-([N-(2- iodophenyl)phenylsulfonamido]methyl)-1-phenylsulfonyl-1*H*-indole-3- carboxylate (Senthil Kumar, *et al.*, 2006). Bond lengths and angles (S1—O1 =1.427 (1) Å, S1—O2 = 1.431 (1) Å, O1-S1-O2 = 121.3 (1) °, O1-S1-N1 = 107.3 (9) °, O2S1N1 = 104.7 (1) °) involving the S atom of the phenylsufonyl group present in the molecule is similar to the distances (S1—O1 = 1.425 Å, S1—O2 = 1.429 Å) and angles (O1-S1-O2 = 120.4 (8) °, O1-S1-N1 = 106.9 (7) °, O2-S1-N1 = 106.7 (7) °)) that reported in the literature (Senthil Kumar, *et al.*, 2006); the O—S—O, N—S—C and N—S—O angles deviate significantly from the ideal tetrahedral value (Table 1), which is consistent to the reported data in the literature (Senthil Kumar, *et al.*, 2006). The phenyl rings (C2—>C7) and (C8—>C13) are planar to within 0.01 Å. The dihedral angle between the two phenyl rings is 52.3 (1) °.

The crystal struture is further stabilized by hydrogen bonding. As shown in Fig.2, a dimeric structure is formed *via* intermolecular hydrogen bonds N1—H2···O3^i^ (i = 1 - *x*, 1 - *y*, -*z*) (Table 2).

### Experimental

The mixture of *N*-(2-formylphenyl)(4-methylbenzene)sulfonamide (0.500 g, 1.82 mmol) and (ethoxycarbonylmethylene)triphenylphosphorane (0.700 g, 2.00 mmol) in dichloromethane (10 ml) was stirred at room temperature for 2 h (Scheme 2). After evaporation of the solvent, the title compound was obtained from the residue by chromatography. Single crystals suitable for X-ray analysis were obtained from ethyl acetate by slow evaporation.

### Refinement

All H atoms were fixed geometrically and treated as riding with C—H = 0.93-0.96 Å and N—H = 0.92 Å, and *U*_iso_(H) = 1.2-1.5 *U*_eq_(host).

### Crystal data

**Table:**

C_18_H_19_NO_4_S	*Z* = 2
*M**_r_* = 345.40	*F*(000) = 364
Triclinic, *P*1	*D*_x_ = 1.314 Mg m^−^^3^
Hall symbol: -P 1	Mo *K*α radiation, λ = 0.71073 Å
*a* = 8.001 (4) Å	Cell parameters from 8607 reflections
*b* = 10.245 (5) Å	θ = 2.8–29.2°
*c* = 11.402 (5) Å	µ = 0.21 mm^−^^1^
α = 81.182 (5)°	*T* = 291 K
β = 70.895 (4)°	Block, colourless
γ = 86.604 (5)°	0.46 × 0.43 × 0.38 mm
*V* = 872.7 (8) Å^3^	

### Data collection

**Table:**

Oxford Diffraction Gemini S Ultra diffractometer	3553 independent reflections
Radiation source: fine-focus sealed tube	2784 reflections with *I* > 2σ(*I*)
graphite	*R*_int_ = 0.020
Detector resolution: 15.9149 pixels mm^-1^	θ_max_ = 26.4°, θ_min_ = 2.8°
ω scans	*h* = −9→9
Absorption correction: multi-scan (*SADABS*; Bruker, 2001)	*k* = −12→12
*T*_min_ = 0.91, *T*_max_ = 0.93	*l* = −14→14
15343 measured reflections	

### Refinement

**Table:**

Refinement on *F*^2^	Primary atom site location: structure-invariant direct methods
Least-squares matrix: full	Secondary atom site location: difference Fourier map
*R*[*F*^2^ > 2σ(*F*^2^)] = 0.044	Hydrogen site location: inferred from neighbouring sites
*wR*(*F*^2^) = 0.147	H-atom parameters constrained
*S* = 1.18	*w* = 1/[σ^2^(*F*_o_^2^) + (0.0903*P*)^2^] where *P* = (*F*_o_^2^ + 2*F*_c_^2^)/3
3553 reflections	(Δ/σ)_max_ < 0.001
219 parameters	Δρ_max_ = 0.24 e Å^−^^3^
0 restraints	Δρ_min_ = −0.28 e Å^−^^3^

### Special details

**Table:**

Geometry. All esds (except the esd in the dihedral angle between two l.s. planes) are estimated using the full covariance matrix. The cell esds are taken into account individually in the estimation of esds in distances, angles and torsion angles; correlations between esds in cell parameters are only used when they are defined by crystal symmetry. An approximate (isotropic) treatment of cell esds is used for estimating esds involving l.s. planes.
Refinement. Refinement of *F*^2^ against ALL reflections. The weighted *R*-factor *wR* and goodness of fit *S* are based on *F*^2^, conventional *R*-factors *R* are based on *F*, with *F* set to zero for negative *F*^2^. The threshold expression of *F*^2^ > σ(*F*^2^) is used only for calculating *R*-factors(gt) *etc*. and is not relevant to the choice of reflections for refinement. *R*-factors based on *F*^2^ are statistically about twice as large as those based on *F*, and *R*- factors based on ALL data will be even larger.

### Fractional atomic coordinates and isotropic or equivalent isotropic displacement parameters (Å^2^)

**Table:**

	*x*	*y*	*z*	*U*_iso_*/*U*_eq_	
S1	0.72847 (6)	0.25225 (4)	0.25492 (5)	0.0646 (2)	
O1	0.70944 (18)	0.19430 (14)	0.38058 (14)	0.0831 (5)	
O2	0.86898 (18)	0.21412 (15)	0.15094 (16)	0.0947 (5)	
O3	0.4015 (2)	0.67440 (14)	0.03433 (11)	0.0782 (4)	
O4	0.2137 (2)	0.78869 (13)	0.17227 (12)	0.0891 (5)	
N1	0.54807 (18)	0.22083 (13)	0.22784 (12)	0.0526 (4)	
H1	0.5561	0.2476	0.1454	0.063*	
C1	0.7190 (4)	0.8460 (2)	0.2182 (3)	0.1029 (8)	
H1A	0.8038	0.8833	0.1408	0.154*	
H1B	0.7451	0.8732	0.2872	0.154*	
H1C	0.6024	0.8761	0.2198	0.154*	
C2	0.7276 (3)	0.69829 (19)	0.22896 (18)	0.0662 (5)	
C3	0.7857 (3)	0.6372 (2)	0.12236 (19)	0.0740 (6)	
H3	0.8228	0.6888	0.0440	0.089*	
C4	0.7895 (3)	0.5024 (2)	0.12980 (18)	0.0690 (5)	
H4	0.8275	0.4629	0.0571	0.083*	
C5	0.7361 (2)	0.42488 (17)	0.24668 (16)	0.0548 (4)	
C6	0.6821 (2)	0.4838 (2)	0.35380 (17)	0.0640 (5)	
H6	0.6484	0.4326	0.4324	0.077*	
C7	0.6786 (3)	0.6198 (2)	0.34324 (19)	0.0706 (5)	
H7	0.6419	0.6595	0.4158	0.085*	
C8	0.3783 (2)	0.22505 (15)	0.32128 (13)	0.0459 (4)	
C9	0.3306 (2)	0.11643 (16)	0.41448 (14)	0.0533 (4)	
H9	0.4105	0.0470	0.4163	0.064*	
C10	0.1657 (2)	0.11117 (18)	0.50398 (15)	0.0598 (5)	
H10	0.1346	0.0386	0.5663	0.072*	
C11	0.0473 (2)	0.21322 (19)	0.50108 (16)	0.0643 (5)	
H11	−0.0647	0.2091	0.5609	0.077*	
C12	0.0937 (2)	0.32127 (18)	0.41022 (16)	0.0578 (4)	
H12	0.0122	0.3897	0.4095	0.069*	
C13	0.2609 (2)	0.33082 (15)	0.31862 (13)	0.0461 (4)	
C14	0.3083 (2)	0.44882 (16)	0.22402 (14)	0.0494 (4)	
H14	0.4004	0.4398	0.1505	0.059*	
C15	0.2336 (3)	0.56488 (18)	0.23341 (15)	0.0671 (5)	
H15	0.1397	0.5761	0.3052	0.080*	
C16	0.2919 (3)	0.67872 (17)	0.13510 (15)	0.0592 (5)	
C17	0.2604 (4)	0.9087 (2)	0.08528 (19)	0.1009 (9)	
H17A	0.3855	0.9073	0.0380	0.121*	
H17B	0.1945	0.9160	0.0266	0.121*	
C18	0.2205 (4)	1.0191 (2)	0.1528 (2)	0.0906 (7)	
H18A	0.2421	1.0994	0.0947	0.136*	
H18B	0.2941	1.0158	0.2049	0.136*	
H18C	0.0985	1.0162	0.2042	0.136*	

### Atomic displacement parameters (Å^2^)

**Table:**

	*U*^11^	*U*^22^	*U*^33^	*U*^12^	*U*^13^	*U*^23^
S1	0.0528 (3)	0.0488 (3)	0.0846 (4)	0.0071 (2)	−0.0191 (2)	0.0037 (2)
O1	0.0854 (10)	0.0685 (9)	0.1021 (11)	−0.0082 (7)	−0.0561 (9)	0.0283 (8)
O2	0.0584 (8)	0.0700 (10)	0.1324 (13)	0.0181 (7)	−0.0011 (8)	−0.0179 (9)
O3	0.1002 (10)	0.0654 (9)	0.0459 (7)	0.0207 (7)	−0.0027 (7)	0.0068 (6)
O4	0.1339 (13)	0.0402 (7)	0.0584 (8)	0.0133 (7)	0.0093 (8)	0.0022 (6)
N1	0.0575 (8)	0.0454 (7)	0.0484 (7)	0.0050 (6)	−0.0125 (6)	0.0007 (6)
C1	0.153 (2)	0.0586 (13)	0.1080 (18)	−0.0072 (14)	−0.0580 (17)	−0.0062 (13)
C2	0.0749 (12)	0.0547 (11)	0.0725 (12)	−0.0066 (9)	−0.0297 (10)	−0.0035 (9)
C3	0.0894 (14)	0.0598 (12)	0.0637 (11)	−0.0128 (10)	−0.0193 (10)	0.0107 (9)
C4	0.0772 (13)	0.0585 (11)	0.0595 (11)	−0.0064 (9)	−0.0077 (9)	−0.0030 (9)
C5	0.0429 (9)	0.0537 (10)	0.0604 (10)	−0.0024 (7)	−0.0112 (7)	0.0031 (8)
C6	0.0629 (11)	0.0661 (12)	0.0553 (10)	−0.0075 (9)	−0.0140 (8)	0.0063 (9)
C7	0.0790 (13)	0.0664 (13)	0.0641 (11)	−0.0047 (10)	−0.0171 (10)	−0.0138 (9)
C8	0.0542 (9)	0.0409 (8)	0.0429 (8)	−0.0037 (7)	−0.0170 (7)	−0.0030 (6)
C9	0.0689 (11)	0.0417 (9)	0.0497 (9)	−0.0031 (8)	−0.0232 (8)	0.0023 (7)
C10	0.0731 (12)	0.0512 (10)	0.0521 (9)	−0.0173 (9)	−0.0202 (9)	0.0082 (7)
C11	0.0575 (10)	0.0652 (12)	0.0594 (10)	−0.0140 (9)	−0.0078 (8)	0.0042 (9)
C12	0.0506 (9)	0.0557 (10)	0.0614 (10)	−0.0013 (8)	−0.0151 (8)	0.0025 (8)
C13	0.0504 (9)	0.0438 (9)	0.0433 (8)	−0.0033 (7)	−0.0169 (7)	0.0008 (6)
C14	0.0536 (9)	0.0459 (9)	0.0429 (8)	0.0040 (7)	−0.0121 (7)	0.0013 (6)
C15	0.0839 (13)	0.0472 (10)	0.0487 (9)	0.0063 (9)	0.0029 (9)	0.0008 (8)
C16	0.0737 (12)	0.0467 (10)	0.0475 (9)	0.0056 (8)	−0.0093 (8)	−0.0025 (7)
C17	0.166 (2)	0.0426 (11)	0.0665 (13)	−0.0021 (13)	−0.0062 (14)	0.0073 (9)
C18	0.128 (2)	0.0522 (12)	0.0851 (15)	−0.0036 (12)	−0.0264 (14)	−0.0070 (11)

### Geometric parameters (Å, °)

**Table:**

S1—O1	1.4268 (15)	C7—H7	0.9300
S1—O2	1.4282 (15)	C8—C9	1.391 (2)
S1—N1	1.6308 (16)	C8—C13	1.395 (2)
S1—C5	1.760 (2)	C9—C10	1.377 (2)
O3—C16	1.201 (2)	C9—H9	0.9300
O4—C16	1.315 (2)	C10—C11	1.373 (3)
O4—C17	1.440 (2)	C10—H10	0.9300
N1—C8	1.429 (2)	C11—C12	1.372 (2)
N1—H1	0.9203	C11—H11	0.9300
C1—C2	1.499 (3)	C12—C13	1.400 (2)
C1—H1A	0.9600	C12—H12	0.9300
C1—H1B	0.9600	C13—C14	1.468 (2)
C1—H1C	0.9600	C14—C15	1.302 (2)
C2—C7	1.371 (3)	C14—H14	0.9300
C2—C3	1.383 (3)	C15—C16	1.468 (2)
C3—C4	1.371 (3)	C15—H15	0.9300
C3—H3	0.9300	C17—C18	1.427 (3)
C4—C5	1.390 (2)	C17—H17A	0.9700
C4—H4	0.9300	C17—H17B	0.9700
C5—C6	1.376 (3)	C18—H18A	0.9600
C6—C7	1.380 (3)	C18—H18B	0.9600
C6—H6	0.9300	C18—H18C	0.9600
			
O1—S1—O2	121.53 (10)	C10—C9—C8	120.32 (16)
O1—S1—N1	107.04 (8)	C10—C9—H9	119.8
O2—S1—N1	104.81 (9)	C8—C9—H9	119.8
O1—S1—C5	108.14 (9)	C11—C10—C9	119.93 (16)
O2—S1—C5	107.81 (8)	C11—C10—H10	120.0
N1—S1—C5	106.63 (7)	C9—C10—H10	120.0
C16—O4—C17	117.49 (15)	C12—C11—C10	120.17 (17)
C8—N1—S1	121.54 (11)	C12—C11—H11	119.9
C8—N1—H1	118.1	C10—C11—H11	119.9
S1—N1—H1	111.8	C11—C12—C13	121.51 (17)
C2—C1—H1A	109.5	C11—C12—H12	119.2
C2—C1—H1B	109.5	C13—C12—H12	119.2
H1A—C1—H1B	109.5	C8—C13—C12	117.60 (14)
C2—C1—H1C	109.5	C8—C13—C14	122.03 (14)
H1A—C1—H1C	109.5	C12—C13—C14	120.38 (15)
H1B—C1—H1C	109.5	C15—C14—C13	126.10 (15)
C7—C2—C3	118.06 (19)	C15—C14—H14	116.9
C7—C2—C1	121.61 (19)	C13—C14—H14	116.9
C3—C2—C1	120.33 (18)	C14—C15—C16	122.61 (16)
C4—C3—C2	121.34 (18)	C14—C15—H15	118.7
C4—C3—H3	119.3	C16—C15—H15	118.7
C2—C3—H3	119.3	O3—C16—O4	123.41 (17)
C3—C4—C5	119.55 (19)	O3—C16—C15	124.92 (17)
C3—C4—H4	120.2	O4—C16—C15	111.63 (15)
C5—C4—H4	120.2	C18—C17—O4	109.33 (17)
C6—C5—C4	119.91 (17)	C18—C17—H17A	109.8
C6—C5—S1	120.91 (13)	O4—C17—H17A	109.8
C4—C5—S1	119.11 (14)	C18—C17—H17B	109.8
C5—C6—C7	119.18 (17)	O4—C17—H17B	109.8
C5—C6—H6	120.4	H17A—C17—H17B	108.3
C7—C6—H6	120.4	C17—C18—H18A	109.5
C2—C7—C6	121.93 (18)	C17—C18—H18B	109.5
C2—C7—H7	119.0	H18A—C18—H18B	109.5
C6—C7—H7	119.0	C17—C18—H18C	109.5
C9—C8—C13	120.45 (15)	H18A—C18—H18C	109.5
C9—C8—N1	117.27 (15)	H18B—C18—H18C	109.5
C13—C8—N1	122.25 (13)		

### Hydrogen-bond geometry (Å, °)

**Table:**

*D*—H···*A*	*D*—H	H···*A*	*D*···*A*	*D*—H···*A*
N1—H1···O3^i^	0.92	2.01	2.920 (2)	172

Symmetry codes: (i) −*x*+1, −*y*+1, −*z*.

## References

[bb1] Bruker (2001). *SADABS* Bruker AXS Inc., Madison, Wisconsin, USA.

[bb2] Farrugia, L. J. (1997). *J. Appl. Cryst.* **30**, 565.

[bb3] Farrugia, L. J. (1999). *J. Appl. Cryst.* **32**, 837–838.

[bb4] Mukherjee, S., Yang, J. W., Hoffmann, S. & List, B. (2007). *Chem. Rev.* **107**, 5471–5569. 10.1021/cr0684016 18072803

[bb5] Oxford Diffraction (2006). *CrysAlis Pro* Oxford Diffraction Ltd, Abingdon, England.

[bb6] Patchett, A. A., Nargund, R. P. & Tata, J. R. (1995). *Proc. Natl Acad. Sci. USA*, **92**, 7001–7005. 10.1073/pnas.92.15.7001 PMC41459 7624358

[bb7] Pearce, L. J. & Watkin, D. J. (1993). *CAMERON* Chemical Crystallography Laboratory, University of Oxford, England.

[bb8] Senthil Kumar, G., Chinnakali, K., Ramesh, N., Mohanakrishnan, A. K. & Fun, H.-K. (2006). *Acta Cryst.* E**62**, o5905–o5907.

[bb9] Sheldrick, G. M. (2008). *Acta Cryst.* A**64**, 112–122. 10.1107/S0108767307043930 18156677

